# Effect of Curcumin on the Diversity of Gut Microbiota in Ovariectomized Rats

**DOI:** 10.3390/nu9101146

**Published:** 2017-10-19

**Authors:** Zhiguo Zhang, Yanjing Chen, Lihua Xiang, Zhen Wang, Gary Guishan Xiao, Jingqing Hu

**Affiliations:** 1 Institute of Basic Theory, China Academy of Chinese Medical Sciences, Beijing 100700, China; zzgtcm@163.com (Z.Z.); chenyj010@163.com (Y.C.); xlh891201@sina.com (L.X.); wangzhenbj@163.com (Z.W.); 2 School of Pharmaceutical Science, Dalian University of Technology, Dalian 116024, China

**Keywords:** menopause, ovariectomy, rat, curcumin, gut microbiota

## Abstract

Curcumin has been proven to have a weight-loss effect in a menopausal rat model induced by ovariectomy. However, the effects of curcumin on gut microfloral communities of ovariectomized (OVX) rats remains unclear. Here, we used high-throughput 16S rDNA sequencing to explore the effects of curcumin on microbial diversity in the gut of OVX rats. Female Wistar rats were subjected to either ovariectomy or a sham operation (SHAM group). The OVX rats were treated with vehicle (OVX group) or curcumin (CUR group) by oral gavage. After 12-week treatments, the weights of the bodies and uteri of rats were recorded, the levels of estradiol in the serum were assayed by electrochemiluminescence immunoassay (ECLIA). Then, the fragments encompassing V3–V4 16S rDNA hypervariable regions were PCR amplified from fecal samples, and the PCR products of V3–V4 were sequenced on an Illumina MiSeq for characterization of the gut microbiota. Our results showed that, compared to rats in the SHAM group, rats in the OVX group had more weight gain and lower levels of estradiol in the serum, and curcumin could cause significant weight loss in OVX rats but did not increase the levels of estradiol. Sequencing results revealed the presence of 1120, 1114, and 1119 operational taxonomic units (OTUs) found in the SHAM, OVX, and CUR groups, respectively. The percentage of shared OTUs was 86.1603%. Gut microbiota of rats from the SHAM or CUR group had higher levels of biodiversity and unevenness estimations than those from the OVX group. At the phyla level, compared to rats in SHAM group, rats in the OVX group had a higher ratio of phyla *Firmicutes* and *Bacteroidetes* in the gut; at the genus level, four differential gut microbiota (*Incertae_Sedis*, *Anaerovorax*, *Anaerotruncus*, and *Helicobacter*) between SHAM and OVX groups were found, whereas seven differential gut microbiota (*Serratia*, *Anaerotruncus*, *Shewanella*, *Pseudomonas*, *Papillibacter*, *Exiguobacterium*, and *Helicobacter*) between OVX and CUR groups were found. In conclusion, estrogen deficiency induced by ovariectomy caused changes in the distribution and structure of intestinal microflora in rats, and curcumin could partially reverse changes in the diversity of gut microbiota.

## 1. Introduction

Menopause is a natural process that all women will go through, usually accompanied by some physiological and psychological changes, including bone loss, weight gain, depression, nervousness, etc. [1,2,3]. Huge quantities of microfloral communities in the gut play an important role in nutrient absorbance, pathogen defense, immune response, and energy metabolism [4,5,6]. Several studies showed that microfloral communities in the guts of menopausal women or animal models also had significant changes, and some drugs or foods had modulatory effects on these changes [7,8,9]. 

Curcumin is an active component of turmeric (*Curcuma longa*), used as a spice and a traditional medicine for centuries in Asian countries [10]. Its anti-inflammatory, antimutagen, antioxidant, and anti-infectious properties have been previously studied [11,12,13,14]. In a menopausal rat model induced by ovariectomy, curcumin had been widely proven to have weight-loss effects [15,16]. However, what effects curcumin exerts on microfloral communities in the intestines of ovariectomized (OVX) rats remain unclear. To answer this question, in the present study, we explored the effect of curcumin on the diversity of gut microbiota in OVX rats for the first time. 

## 2. Materials and Methods

### 2.1. Animals

In this study, 18 virgin Wistar rats aged six months and weighing approximately 310 ± 20.0 g were acquired from the Experimental Animal Center in the Academy of Military Medical Sciences (SCXK-(Military) 2016-006, Beijing, China). The protocol involving animals in this study was authorized by The Institutional Ethics Committee of China Academy of Chinese Medical Sciences (Approval number: 2016-017). The sham operation (*n* = 6, SHAM group) or bilateral OVX (*n* = 12) using a dorsal incision as we reported previously [17] was performed on the rats after acclimatization. The rats undergoing OVX were divided into two groups based on the treatment agents, including vehicle (OVX group, *n* = 6) and curcumin (CUR group, *n* = 6). The rats in the CUR group were administered curcumin (Sigma-Aldrich, Saint Louis, MO, USA, dissolved in distilled water) at 100 mg/kg/day by oral gavage. The rats in the SHAM and the OVX groups were administered the same volume of distilled water by oral gavage. All rats were fed standard chow during the course of the experiments (Animal Center of the Fourth Military Medical University, Xi’an, China). The treatment started one week after surgery for 12 weeks. The body weight of each rat was monitored weekly.

### 2.2. Preparation of Specimens

The day after the last treatment, the animals were anesthetized with an intraperitoneal injection of ketamine (80 mg/kg body weight) and xylazine (12 mg/kg body weight) and sacrificed by exsanguination. The uterus was dissected out and immediately weighed. Blood samples were obtained by puncturing the abdominal aorta before death and were collected in tubes. Then, serum was prepared by centrifugation (3000 rpm for 10 min) and preserved at −80 °C.

### 2.3. Measurements of Estradiol in Serum

The estradiol concentration in the serum was detected using an electrochemiluminescence immunoassay (ECLIA) assay kit (Roche Diagnostics, Mannheim, Germany), in accordance with the instructions. All measurements were performed according to the manufacturers’ protocols.

### 2.4. Fecal Collection and Bacterial DNA Extraction

Rat colons were immediately excised, and fecal samples were harvested for microbial DNA extraction using E.Z.N.A.^®^ Stool DNA Kit (Omega Bio-tek, Norcross, GA, USA), following the manufacturer’s instructions. The quality and quantity of genomic DNA were assessed with a nanodrop spectrophotometer, with the A260/A280 ratio between 1.8 and 2.0 considered a criterion for quality control. No obvious RNA banding was shown by gel electrophoresis, and genomic bands were clear and complete. DNA was frozen at −80 °C prior to PCR amplification.

### 2.5. PCR Amplification and Sequencing

Fragments encompassing V3–V4 16S rDNA hypervariable regions were PCR amplified from each of the 18 DNA samples using fusion primers (forward: 5′-barcode-GTGCCAGCMGCCGCGG-3′, reverse: 5′-CCGTCAATTCMTTTRAGTTT-3′) and universal primers (forward: 5′-AYTGGGYDTAAAGNG-3′, reverse: 5′-TACNVGGGTATCTAATCC-3′).

The PCR conditions were as follows: 95 °C for 2 min, 25 cycles of 95 °C for 30 s, 55 °C for 30 s and 72 °C for 30 s, 72 °C for 5 min and holding at 4 °C. PCR products were excisedfrom 1% agarose gels and purified using an AxyPrep DNA GelExtraction Kit (Axygen Biosciences, Union City, CA, USA). The V3–V4 PCR products were sequenced by Illumina MiSeq (Illumina, San Diego, CA, USA).

### 2.6. Statistical Analysis

Part of quantitative data (weights of bodies and uteri, and level of estradiol in serum) are reported as the mean ± standard deviation; the proportion of gut microbiota is reported as the mean ± standard error. Statistical differences in basal characteristics between the groups were calculated by one-way analysis of variance and *q* test for continuous variables. *p* < 0.05 was considered statistically significant. All statistical analyses were performed using the SPSS 19.0 software (IBM Corporation, Somers, NY, USA).

## 3. Results

### 3.1. The Effect of Curcumin on the Weights of Bodies and Uteri

**Figure 1:**
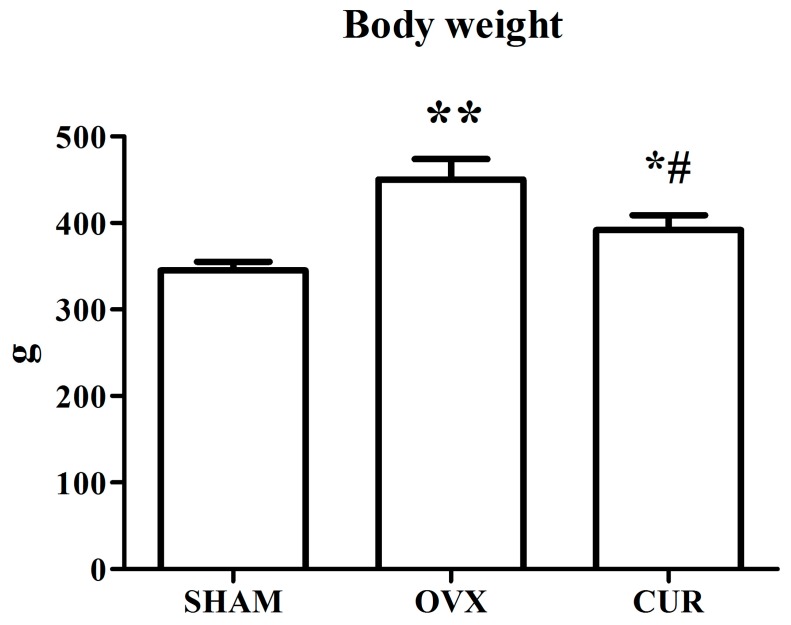
The effect of curcumin on body weight after 12-week treatment. ** *p* < 0.01 versus SHAM group, * *p* < 0.05 versus SHAM group, ^#^ *p* < 0.01 versus OVX group.

**Figure 2:**
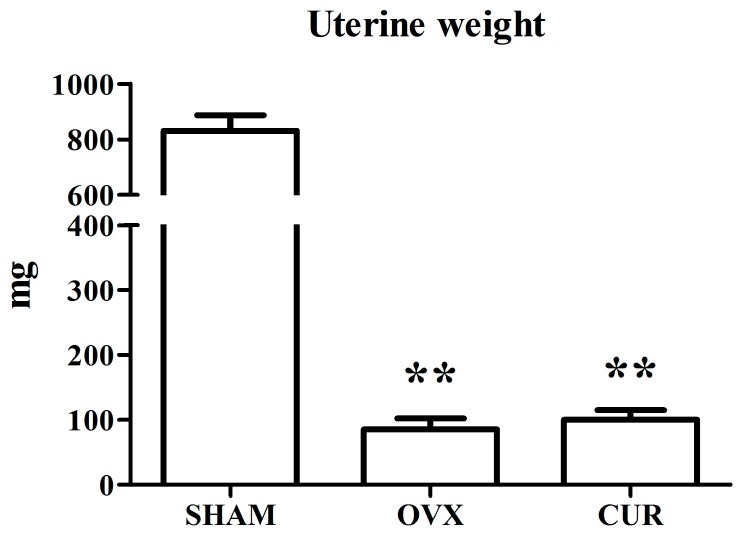
The effect of curcumin on uterine weight after 12-week treatment. ** *p* < 0.01 versus SHAM group.

Figure 1 shows that the SHAM rats weighted significantly less compared to the OVX rats. Curcumin could inhibit weight gain induced by OVX during the 12-week treatment. Compared to the SHAM group, OVX caused remarkable atrophy of the uterus, indicating a successful operation. The administration of curcumin had no remarkable anti-atrophic effect on uterine tissue compared to the OVX group rats (Figure 2).

### 3.2. The Effect of Curcumin on the Level of Estradiol in Serum

**Figure 3:**
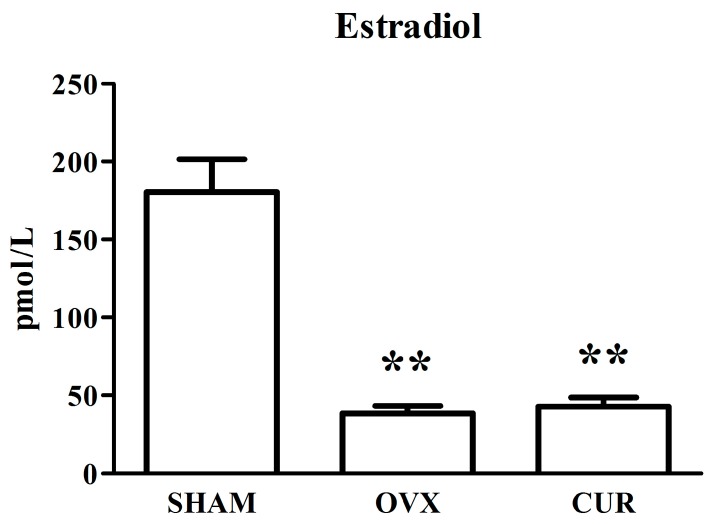
The effect of curcumin on estradiol levels in serum after 12-week treatment. ** *p* < 0.01 versus SHAM group.

Figure 3 shows the serum concentrations of estradiol in different rats after treatment for 12 weeks. After the 12-week treatment, the levels of estradiol in the SHAM rats were higher in comparison to the OVX rats (*p* < 0.01). In addition, the levels of estradiol in the CUR group rats had no significant change in comparison to the OVX group rats.

### 3.3. Quality Evaluation and Filtering of Original Data

Pandaseq software [18] was used to control the quality of the raw data by truncating or abandoning low-quality sequences. The ends of the corresponding sequences were connected. The sequences that were unable to connect were abandoned. According to the experimental requirements, the connected sequences were filtered for analysis. A total of 1,242,181 V3–V4 16S rDNA sequence reads from the 18fecal samples, with an average of 69,010 sequence reads for each fecal sample (the minimum numbers of reads per sample were 45,099), were used in this analysis. The average length of the sequence reads was 225 bp, and they were classified into different taxonomic categories.

### 3.4. Operational Taxonomic Units

**Figure 4:**
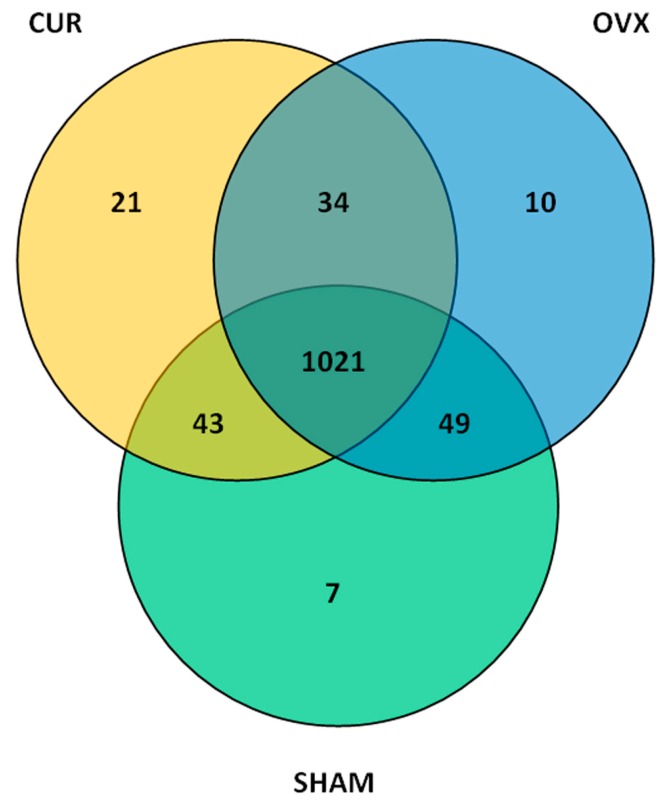
The number of differential OTUs in fecal samples after 12-week treatment of curcumin. Venn diagrams indicate the number of overlapping differential OTUs between fecal samples from three groups.

According to the sequence similarity (>97%), high quality sequences were classified into multiple operational taxonomic units (OTUs) using QIIME (version 1.8.0) (http://qiime.org/) [19] to facilitate analysis. The OTU in each fecal sample and the number of sequences in each OTU were counted to obtain the taxonomic information of the OTU. The taxon abundance of each fecal sample was generated into 12 phyla, 20 classes, 26 orders, 39 families, and 54 genera using mainly the RDP, Greengenes, and SSU databases. A total of 1120, 1114, and 1119 OTUs were found in the SHAM, OVX, and CUR groups, respectively. The three groups shared 1021 (86.1603%) OTUs, and the total richness for all groups was 1185 (Figure 4).

### 3.5. Alpha Diversity Analysis

Alpha diversity refers to the diversity in a specific area or ecosystem in terms of species richness. According to species richness in the list of OTUs in the fecal sample, diversity, richness, coverage, and evenness estimations were calculated for all data sets using Mothur Software (https://www.mothur.org/). The Chao or Ace calculation is an estimator of phylotype richness, and the Shannon or Simpson index of diversity reflects both the richness and community evenness. The OVX group had a lower richness index (Chao, 746; and ACE, 825.70) and higher evenness index (Shannon, 7.16; and Simpson, 0.980) than the SHAM group (Chao, 776; ACE, 878.71; Shannon, 7.00; and Simpson, 0.972) and CUR group (Chao, 762; ACE, 847.71; Shannon, 7.00; and Simpson, 0.975). These results suggested that the OVX group had lower level of biodiversity and higher evenness than the SHAM group and CUR group (Table 1).

### 3.6. Analysis of Differential Gut Microbiota

**Figure 5:**
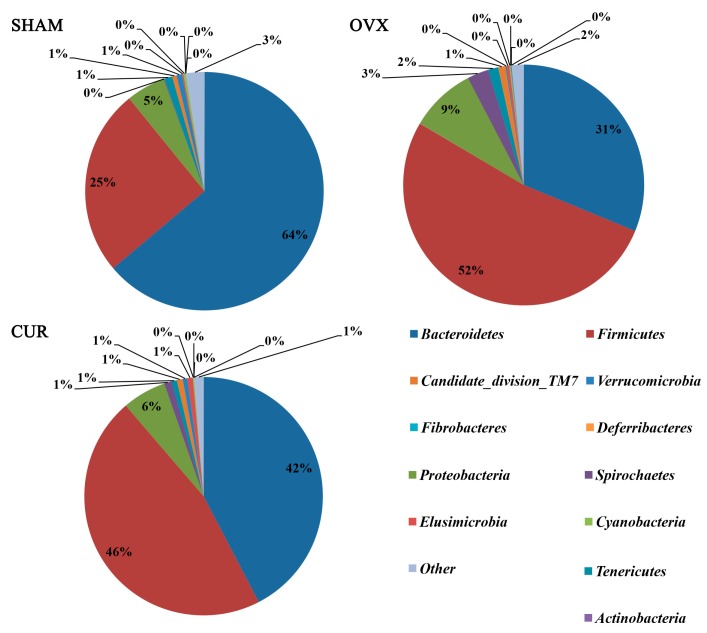
Microbial distributions at the phylum level in the fecal samples from the three groups. Percentages are based on proportions of assignable reads.

**Figure 6:**
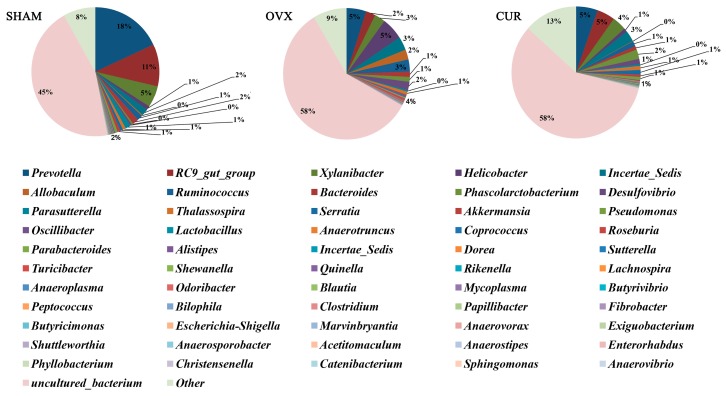
Microbial distributions at the genus level in the fecal samples from the three groups. Percentages are based on proportions of assignable reads.

Twelve phyla and 56 genera in total were found between the three groups. At the phylum level, a significant difference in the number of species of *Firmicutes* and *Bacteroidetes* was found between the OVX and SHAM group (Table 2), but no significant difference in the number of species was found between the CUR and OVX group. At the genus level, we found fourgenera (*Incertae_Sedis*, *Anaerovorax*, *Anaerotruncus*, *Helicobacter*) that had significant differences in the number of species between the OVX and SHAM group (Table 3), and seven genera (*Serratia*, *Anaerovorax*, *Shewanella*, *Pseudomonas*, *Papillibacter*, *Exiguobacterium*, *Helicobacter*) that had significant differences in the number of species between the CUR and OVX group (Table 4). Figure 5 and Figure 6 showed microbial distributions at the phylum and genus level in the fecal samples from the three groups.

### 3.7. Beta Diversity Analysis

**Figure 7:**
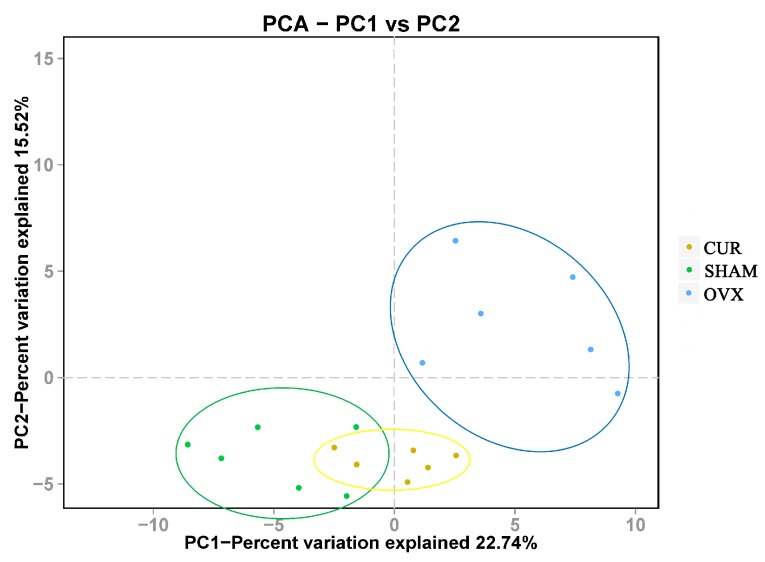
Clustering of microbial communities using principal component analysis from 18 fecal samples. The percentage of variation explained by the principal component is indicated on the axes. Subject color coding: blue, fecal samples from the OVX group; green, fecal samples from the SHAM group; yellow, fecal samples from the CUR group.

Beta diversity analysis represents the extent of similarity between different microbial communities. Two principal components were extracted by principal component analysis (PCA), and scatter plots were generated in Origin 8.0. Each point indicates the position of each sample and the entries are distributed according to their relatedness. Figure 7 showed a clear separation between the fecal samples from SHAM, OVX, and CUR groups. In particular, the fecal samples from the OVX group were well separated from those from the SHAM or CUR group. Percentage values at the axes indicate contribution of the principal components to the explanation of total variance in the dataset. The figure showed that the percentages of variation explained byPC1 and PC2 were 22.74% and 15.52%, respectively.

## 4. Discussion

In the current study, compared to SHAM rats, ovariectomy led to significant body weight gain and loss of uterine wet weight in the OVX group rats. However, curcumin had a significant preventive effect on body weight gain rather than loss of uterine wet weight of OVX rats (Figure 1 and Figure 2). In addition, the results from the ECLIA assay showed that the serum estradiol levels of the SHAM group rats were higher in comparison to the OVX and CUR group rats, which coincided with previous reports [20,21]. We inferred that CUR had no estrogen-like action but could be a potential weight loss agent for menopausal women. 

Overweight or obesity is a common phenomenon in menopausal women [22] or rodent models induced by ovariectomy [23]. The reasons for increasing obesity in menopausal women are still unclear. Some researchers hold that the deficiency of estrogen may be an important obesity-triggering factor [24]. More and more evidence suggests that the alteration of the microbial community in the intestine may play an important role in the occurrence of obesity [25], including obesity of menopausal women [26]. Particularly, *Firmicutes* and *Bacteroidetes*, two major phyla of gut microbiota, are thought to be involved in lipid and bile acid metabolism to maintain systemic energy homeostasis in hosts [27,28,29]. It had been proven that an increase in the ratio of *Firmicutes* and *Bacteroidetes* was correlated with the development of obesity in humans or animal models [30,31,32]. In the present study, compared to SHAM rats, the abundance of *Firmicutes* and *Bacteroidetes* in the gut of the OVX group were significantly increased and decreased, respectively. We speculated that the increased *Firmicutes*/*Bacteroidetes* ratio plays an important role in overweight OVX rats. 

At the genus level, compared to OVX group rats, the abundance of *Incertae_Sedis*, *Anaerovorax*, *Anaerotruncus*, and *Helicobacter* in the gut of the SHAM group rats, and the abundance of *Serratia*, *Anaerotruncus*, *Shewanella*, *Pseudomonas*, *Papillibacter*, *Exiguobacterium*, and *Helicobacter* in the gut of the CUR group rats changed significantly. Some studies suggested that an elevated abundance of *Anaerotruncus* in the gut was associated with prenatal stress [33] and age-related macular degeneration [34]. Our results showed that curcumin could decrease the abundance of *Anaerotruncus* in the gut of OVX rats, and we supposed that the studies exploring the effects of CUR on neuropsychic diseases are prospective.

Very little was known about *Helicobacter* in the intestine, and almost all papers were on *Helicobacter pylori*. In murine models of gastric cancer or gastritis induced by *Helicobacter pylori* infection, ovariectomized animals used to have a more aggravated gastric lesion compared to those without ovariectomy [35,36]. Accumulating evidence showed that curcumin was a potent anti-*helicobacter pylori* agent in vivo [37,38] or in vitro [39,40]. In the present study, we found CUR can decrease the abundance of *Helicobacter pylori* in the gut of rats. Further research on the relationship between menopause, curcumin, and *Helicobacter* in the gut is necessary. 

## 5. Conclusions

This study is the first to reveal the effect of curcumin on the diversity of gut microbiota in a menopausal rat model using high-throughput sequencing technology. The results of this study suggested that estrogen deficiency induced by ovariectomy-caused changes in the distribution and structure of intestinal microflora in a rat and that curcumin could partially reverse changes in the diversity of the gut microbiota. At the phyla level, compared to sham rats, model rats had a higher ratio of phyla *Firmicutes* and *Bacteroidetes* in the gut, which may lead to overweight rats; at the genus level, curcumin could lower the increasing abundance of the genera *Anaerotruncus* and *Helicobacter* in the gut of model rats. 

## Figures and Tables

**Table 1:** Estimation of diversity within fecal samples from the three groups.

Group	Chao	Ace	Shannon	Simpson
SHAM	776	878.71	7.00	0.972
OVX	746	825.70	7.16	0.980
CUR	762	847.71	7.00	0.975

**Table 2:** Differential gut microbiota at the phylum level between fecal samples from SHAM and OVX groups ^1^.

Taxa	SHAM	OVX	*p* Value
*Firmicutes*	0.261144 ± 0.054002	0.535624 ± 0.069105	0.009583
*Bacteroidetes*	0.659073 ± 0.068158	0.321806 ± 0.111124	0.036333

^1^ Data are expressed as the means ± SEM (*n* = 6).

**Table 3:** Differential gut microbiota at the genus level between fecal samples from SHAM and OVX groups ^1^.

Taxa	SHAM	OVX	*p* Value
*Incertae_Sedis*	0.043671 ± 0.001527	0.095096 ± 0.005247	0
*Anaerovorax*	0.000233 ± 0.000094	0.001323 ± 0.000079	0.00175
*Anaerotruncus*	0.003101 ± 0.000127	0.006812 ± 0.00127	0.019571
*Helicobacter*	0.027859 ± 0.012836	0.148457 ± 0.051536	0.044554

^1^ Data are expressed as the means ± SEM (*n* = 6).

**Table 4:** Differential gut microbiota at the genus level between fecal samples from OVX and CUR groups ^1^.

Taxa	OVX	CUR	*p* Value
*Serratia*	0.000475 ± 0.00017	0.033828 ± 0.003534	0.0022
*Anaerotruncus*	0.006812 ± 0.00127	0.002321 ± 0.000981	0.004073
*Shewanella*	0 ± 0	0.004168 ± 0.000632	0.005945
*Pseudomonas*	0.000178 ± 0.00009	0.023137 ± 0.005138	0.013782
*Papillibacter*	0.000467 ± 0.000072	0.001236 ± 0.000227	0.028545
*Exiguobacterium*	0.000017 ± 0.000017	0.000988 ± 0.000314	0.032164
*Helicobacter*	0.148457 ± 0.051536	0.027151 ± 0.015317	0.049273

^1^ Data are expressed as the means ± SEM (*n* = 6).
